# Extracellular Vesicles: A More Layer of the Complexity of Hydatid Fluid Contents and Functions

**Published:** 2018

**Authors:** Yixia CHEN, Juntao DING

**Affiliations:** 1. College of Life Science and Engineering, Northwest University for Nationalities, Lanzhou, 730030, China; 2. College of Life Science and Technology, Xinjiang University, Urumqi, P.R. China

## Dear Editor-in-Chief

The discovery of extracellular vesicles (EVs) makes the hydatid fluid (HF) contents more complex. First, EVs are composed of a diversity of active molecules, including proteins, lipids, mRNAs and noncoding RNAs. Although the encapsulated components have yet to be fully deciphered, parasite EVs have been shown to contain a considerable number of different proteins, most of which are absent in the HF (1, 2). More importantly, parasites can secrete nucleic acids especially miRNAs into EVs, demonstrated to be active in modulation of gene expression in receipt cells (3). Second, EV secretion and composition of EV contents are highly dynamic, and show differences under distinct physiological and pathophysiological settings. In addition, different types of cells or tissues secrete EVs harboring different molecules (4).

From these points, the HF EVs should be the mixture of EVs mainly released by hydatid cyst cells, brood capsules and free protoscoleces (1). They are postulated to be distinct at different infectious and developmental stages of *E. granulosus* metacestodes. Third, EVs have two main subtypes-exosomes and ectosomes. The two EV subpopulations show differences in the biogenesis, size and contents. Despite these differences, released exosomes and ectosomes function in a similar way (4).

It is also becoming clear that the EV contents change with the mode of biogenesis. Due to the shedding of ectosomes usually responding to external signals, it is interesting to determine them in the HF in further studies, which will be helpful to uncover the complexity of HF EVs. Fourth, host proteins were reported to be present in preparations of parasite EVs, but they were remarkably different (1), suggesting that they are derived from different sources. The HF is likely to contain host-origin EVs that pass through the hydatid cyst to the HF under unknown mechanisms, and host proteins may be also integrated into parasite EVs during the infection (1). Overall, these factors contribute to the dynamic and complex contents of the HF.

**Fig. 1:**
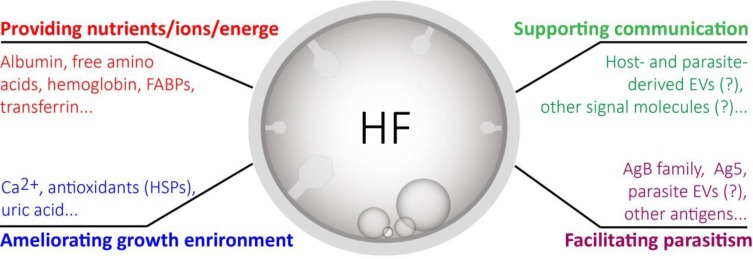
Expanding of HF contents and functions. HF is supposed to be mainly involved in a support of protoscolex growth and development. HF EVs may play a role in intercellular and interorganismal communication as well as parasite pathogenesis. HF: hydatid fluid; EVs: extracellular vesicles; FABPs: fatty acid-binding proteins; HSPs: heat shock proteins; AgB: antigen B; Ag5: antigen 5. ‘?’: functions proposed and further experiments required

Although the functions have not fully understood, the HF is proposed to play a crucial role in supporting protoscolex growth and development (2). Additionally, other functions may exist, such as ameliorating environment. As a number of immune modulators were encapsulated (1), HF EVs might be also involved in parasite-host interactions during the infection (Fig. 1). Furthermore, the presence of EVs indicates the extensive exchanges of active molecules in the HF among cells in the hydatid cyst, protoscoleces, brood capsules and even host cells/tissues.

The discovery of EVs is expanding our knowledge of HF, adding a more layer of the complexity of its contents and functions, and HF EVs give us some clues to understanding of protoscolex growth and development.
